# Identification and Behavioral Evaluation of Sex Pheromone Components of the Chinese Pine Caterpillar Moth, *Dendrolimus tabulaeformis*

**DOI:** 10.1371/journal.pone.0033381

**Published:** 2012-03-13

**Authors:** Xiang-Bo Kong, Kui-Wei Liu, Hong-Bin Wang, Su-Fang Zhang, Zhen Zhang

**Affiliations:** 1 Key Laboratory of Forest Protection, Research Institute of Forest Ecology, Environment and Protection, Chinese Academy of Forestry, State Forestry Administration, Beijing, China; 2 Institute of Plant Protection, Shandong Agricultural University, Tai'an, Shandong, China; INRA-UPMC, France

## Abstract

**Background:**

The Chinese pine caterpillar moth, *Dendrolimus tabulaeformis* Tsai and Liu (Lepidoptera: Lasiocampidae) is the most important defoliator of coniferous trees in northern China. Outbreaks occur over enormous areas and often lead to the death of forests during 2–3 successive years of defoliation. The sex pheromone of *D. tabulaeformis* was investigated to define its chemistry and behavioral activity.

**Methodology/Principal Findings:**

Sex pheromone was collected from calling female *D. tabulaeformis* by headspace solid phase microextraction (SPME) and by solvent extraction of pheromone glands. Extracts were analyzed by coupled gas chromatography/mass spectrometry (GC-MS) and coupled GC-electroantennographic detection (GC-EAD), using antennae from male moths. Five components from the extracts elicited antennal responses. These compounds were identified by a combination of retention indices, electron impact mass spectral matches, and derivatization as (*Z*)-5-dodecenyl acetate (*Z*5-12:OAc), (*Z*)-5-dodecenyl alcohol (*Z*5-12:OH), (5*Z*,7*E*)-5,7-dodecadien-1-yl acetate (*Z*5,*E*7-12:OAc), (5*Z*,7*E*)-5,7-dodecadien-1-yl propionate (*Z*5,*E*7-12:OPr), and (5*Z*,7*E*)-5,7-dodecadien-1-ol (*Z*5,*E*7-12:OH). Behavioral assays showed that male *D. tabulaeformis* strongly discriminated against incomplete and aberrant blend ratios. The correct ratio of *Z*5,*E*7-12:OAc, *Z*5,*E*7-12:OH, and *Z*5,*E*7-12:OPr was essential for optimal upwind flight and source contact. The two monoenes, *Z*5-12:OAc and *Z*5-12:OH, alone or binary mixtures, had no effect on behavioral responses when added to the optimal three-component blend.

**Conclusions/Significance:**

The fact that deviations from the optimal ratio of 100∶100∶4.5 of *Z*5,*E*7-12:OAc, *Z*5,*E*Z7-12:OH, and *Z*5,*E*7-12:OPr resulted in marked decreases in male responses suggests that biosynthesis of the pheromone components is precisely controlled. The optimal blend of the sex pheromone components of *D. tabulaeformis* worked out in this study should find immediate use in monitoring this pest in Chinese forests.

## Introduction

The Chinese pine caterpillar moth, *Dendrolimus tabulaeformis* is native to northern China, where it is an economically important pest of conifers. It is mainly distributed in the provinces of He'bei, He'nan, Shān'xi, Shăn'xi, Shan'dong, Liao'ning, Si'chuan, and Inner Mongolia autonomous region, where it attacks Chinese pine (*Pinus tabulaeformis* Carr.), Scotch pine (*P. sylvestris* var. *mongolica* Litv.), Armand pine (*P. armandii* Franch.), Masson pine (*P. massoniana* Lamb.), and Bunge pine (*P. bungeana* Zucc. ex Endl.) [1]. *Dendrolimus tabulaeformis* has one to three generations per year depending on climatic conditions. In the Beijing region, larvae that eclose from the end of August to mid-September feed until late autumn and then spend the winter in forest litter or soil close to the trunk. In spring, caterpillars climb up to the crown to feed intensively, and pupate in May/June in cocoons. Flights of adults begin in June/July and last until August. Copulation usually lasts ca. 24 h and then females lay eggs on the needles. One egg mass may contain up to several hundred eggs. Control efforts against *D. tabulaeformis* consist mainly of aerial treatment with chemical and bacterial insecticides, and even collection of cocoons during outbreak years. Pheromone-baited traps are used to monitor the population dynamics of several *Dendrolimus* species so that pesticide applications can be optimally timed.

So far, the sex pheromones of six *Dendrolimus* species have been identified, five of which consist of isomers of (5*Z*,7*E*)-dodecadien-1-ol, and/or the corresponding acetates, propionates, or aldehyde derivatives [2]–[8]. The sixth species, *D. houi* uses (5*E*,7*Z*)-dodecadien-1-ol and the corresponding acetate and aldehyde as sex pheromone components [9]. Although preliminary field screening tests showed that a lure impregnated with a 1∶1∶1 ratio of *Z*5,*E*7-12:OH, *Z*5,*E*7-12:OAc, and *Z*5,*E*7-12:OPr attracted a few male *D. tabulaeformis* [10] and the responses of antennae of male *D. tabulaeformis* to these and similar compounds were measured [11], the actual pheromone components of this species and their behavioral roles have not been properly identified. The purpose of this study was to properly identify the sex pheromone of *D. tabulaeformis*. Field and wind tunnel experiments then were carried out to optimize the pheromone blend.

## Results

### GC-EAD and GC analyses of pheromone extracts

**Figure 1:**
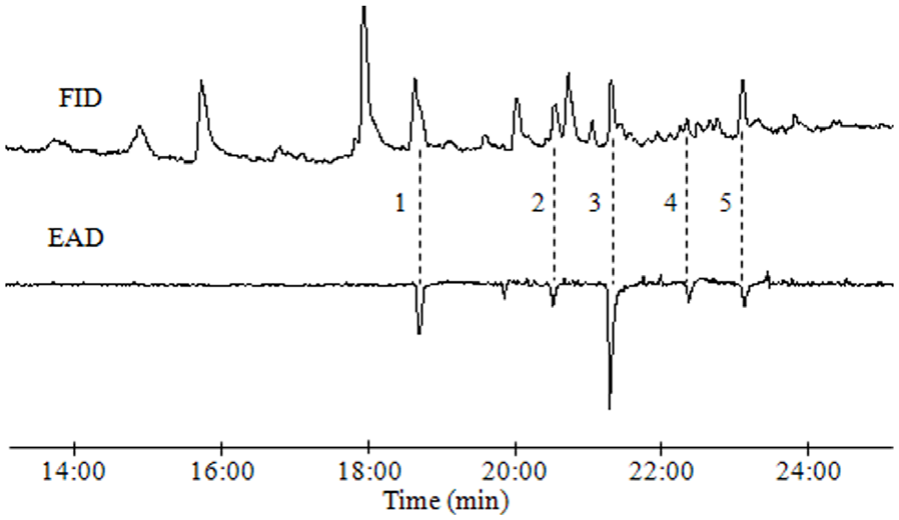
Simultaneously recorded responses from flame ionization detector (FID, top) and electroantennographic detection (EAD, bottom; sensing element: male *D. tabulaeformis* antenna) to headspace volatiles collected by SPME from a virgin calling female *D. tabulaeformis* moth. Peaks indicated by 1–5 represent *Z*5-12:OAc (EAD1), *Z*5-12:OH (EAD2), *Z*5,*E*7-12:OAc (EAD3), *Z*5,*E*7-12:OPr (EAD4), and *Z*5,*E*7-12:OH (EAD5).

GC-EAD analyses of the headspace volatiles emitted by calling females showed five compounds that consistently elicited responses from antennae of male *D. tabulaeformis* moths. These compounds had KIs of 1,899 (EAD1), 2,003 (EAD2), 2,060 (EAD3), 2,126 (EAD4), and 2,180 (EAD5) on a DB-WAX column, corresponding to the KIs and EAD activities of the authentic standards *Z*5-12:OAc, *Z*5-12:OH, *Z*5,*E*7-12:OAc, *Z*5,*E*7-12:OPr, and *Z*5,*E*7-12:OH, respectively (Figure 1, Table 1). Similar results were obtained with GC-EAD analyses of pheromone gland extracts. Thus, the five EAD-active compounds were tentatively identified as *Z*5-12:OAc (EAD1), *Z*5-12:OH (EAD2), *Z*5,*E*7-12:OAc (EAD3), *Z*5,*E*7-12:OPr (EAD4), and *Z*5,*E*7-12:OH (EAD5). In addition, the antennae of male *D. tabulaeformis* also responded to synthetic (5*Z*,7*E*)-5,7-dodecadienal (*Z*5,*E*7-12:Ald) and (5*E*,7*Z*)-5,7-dodecadienal (*E*5,*Z*7-12:Ald).

**Table 1:** Compounds, Kováts Indices, EAD responses, detected rate, and absolute amounts of potential pheromone components collected by CAR/DVB SPME fiber (70 µm) from live calling virgin females of *Dendrolimus tabulaeformis* or extracted from pheromone glands with hexane.

Compounds	Kováts Indices				EAG responses and detected rate				Absolute amounts
	Analyzed by GC-MS on DB-5MS column		Analyzed by GC-EAD on DB-WAX column		Analyzed by GC-EAD on DB-WAX column				Analyzed by GC on DB-WAX column
	standard	SPME collections	Standard	SPME collections	SPME collections		Solvent extracts		Solvent extracts
					Mean (mV)±SD, *N* = 7	Detected (%)	Mean (mV)±SD, *N* = 6	Detected (%)	Mean (ng)±SD, *N* = 21
*Z*5,*E*7-12:OAc	1,635	1,635	2,060	2,060	0.52±0.38	100	0.37±0.15	100	0.47±0.56
*Z*5,*E*7-12:OH	1,509	1,509	2,181	2,180	0.13±0.20	43	0.11±0.11	100	0.49±0.48
*Z*5,*E*7-12:OPr	1,726	1,726	2,126	2,126	0.07±0.08	57	0.13±0.10	100	0.04±0.16
*Z*5-12:OAc	1,588	1,587	1,900	1,899	0.28±0.23	71	0.02±0.03	33	0.19±0.21
*Z*5-12:OH	1,460	1,459	2,004	2,003	0.08±0.11	43	0	0	0.42±0.59

The stereochemistries of the five insect-produced compounds that elicited EAD responses were verified by analyses of extracts on two columns of different polarity (DB-WAX and DB-5MS). On both columns, the retention times of the insect-produced compounds were identical to those of synthetic *Z*5-12:OAc, *Z*5-12:OH, *Z*5,*E*7-12:OAc, *Z*5,*E*7-12:OPr, and *Z*5,*E*7-12:OH, respectively, and markedly different from the KIs of the other isomers. Quantitative analyses on the DB-WAX column showed that single gland solvent extracts contained on average 0.19±0.21 ng (mean ng/female ± SD, *N* = 21) of *Z*5-12:OAc, 0.42±0.59 ng of *Z*5-12:OH, 0.47±0.56 ng of *Z*5,*E*7-12:OAc, 0.04±0.16 ng of *Z*5,*E*7-12:OPr, and 0.49±0.48 ng of *Z*5,*E*7-12:OH, corresponding to a ratio of 39∶86∶96∶8∶100 (Table 1).

### GC-MS analyses of pheromone extracts and MTAD and DMDS adducts

GC-MS analyses of SPME collections on a DB-5MS column confirmed the identities of the five components that had elicited EAD responses, including *Z*5,*E*7-12:OAc (KI: 1,635; M^+•^ at *m*/*z* 224; diagnostic fragment ions: *m*/*z* 164, M^+•^-CH_3_COOH; 136, C_10_H_16_^+^; 121, C_9_H_13_^+^), *Z*5,*E*7-12:OH (KI: 1,509; M^+•^ at *m*/*z* 182; diagnostic fragment ions: *m*/*z* 164, M^+•^-H_2_O; 135, M^+•^-C_2_H_7_O; 121, M^+•^-C_3_H_9_O), *Z*5,*E*7-12:OPr (KI: 1,726; M^+•^ at *m*/*z* 238; diagnostic fragment ions: *m*/*z* 164, M^+•^-C_2_H_5_COOH; 136, C_10_H_16_^+^), *Z*5-12:OAc (KI: 1,587; diagnostic fragment ions: *m*/*z* 166, M^+•^-CH_3_COOH; 138, C_10_H_18_^+^), and *Z*5-12:OH (KI: 1,459; diagnostic fragment ions: *m*/*z* 166, M^+•^-H_2_O) (Table 1 and 2). No other isomers or analogs were detected. The identities of these components were further confirmed by MTAD and DMDS derivatization of gland extracts. Mass spectra and retention times of MTAD derivatized gland extracts (30 FE) were identical with those obtained by MTAD treatment of synthetic *Z*5,*E*7-12:OH (23.50 min), *Z*5,*E*7-12:OAc (24.45 min), and *Z*5,*E*7-12:OPr (25.39 min) (Table 2). The positions of the conjugated double bonds of the components EAD3 (*m*/*z* 337, M^+•^; *m*/*z* 222, (M^+•^-115)), EAD4 (*m*/*z* 351, M^+•^; *m*/*z* 222, (M^+•^-129)), and EAD5 (*m*/*z* 295, M^+•^; *m*/*z* 222, (M^+•^-73)) were confirmed as 5,7 based on the diagnostic fragments from cleavage on either side of the adducts formed between the gland components and the MTAD dienophile (Table 2). GC-MS analysis of the DMDS adducts (30 FE) showed a molecular ion at *m*/*z* 320, a base peak at *m*/*z* 145 [H-(CH_2_)_6_CH = S^+^CH_3_] and a peak at *m*/*z* 175 [CH_3_S^+^ = CH(CH_2_)_4_OOCCH_3_] for EAD1, and a molecular ion at *m*/*z* 278, a base peak at *m*/*z* 145 [H-(CH_2_)_6_CH = S^+^CH_3_] and a peak at *m*/*z* 133 [CH_3_S^+^ = CH(CH_2_)_4_OH] for EAD2, indicating that the double bond was located at position 5 in both compounds (Table 2). The retention times also matched those of the adducts from authentic *Z*5-12:OAc (21.59 min) and *Z*5-12:OH (20.43 min), confirming the previous identifications.

**Table 2:** Electron impact mass spectral data from components of pheromone gland extracts, and their MTAD and DMDS derivatives for the Chinese pine caterpillar, *Dendrolimus tabulaeformis*.

Components/Derivatives	Ions, *m*/*z*/abundance, %^a^				
EAD1	166/53	138/43	123/10	110/46	95/100
EAD2	166/13	138/12	123/6	109/30	67/100
EAD3	224/15	164/31	136/32	121/28	79/100
EAD4	238/12	164/44	136/39	121/21	79/100
EAD5	182/26	164/35	135/8	121/19	79/100
EAD1+DMDS	320/10	213/5	175/9	145/100	115/9
EAD2+DMDS	278/39	213/7	145/100	133/38	85/53
EAD3+MTAD	337/9	280/18	238/13	222/100	165/21
EAD4+MTAD	351/11	294/16	238/23	222/100	165/25
EAD5+MTAD	295/10	238/28	222/100	181/6	165/17

a From full-scan spectra. MTAD, 4-methyl-1,2,4-triazoline-3,5-dione. DMDS, dimethyl disulfide.

### Behavioral assays of the potential pheromone components

Few or no *D. tabulaeformis* males were caught in traps baited with only *Z*5,*E*7-12:OH or *Z*5,*E*7-12:OAc, or binary mixtures of the two (Table 3). Traps baited with *Z*5,*E*7-12:OAc/*Z*5,*E*7-12:OH/*Z*5,*E*7-12:OPr in ratios of 1600∶1600∶0.7 or 1600∶1600∶7 were also no better than unbaited controls. However, traps baited with higher rates of *Z*5,*E*7-12:OPr (70 µg or 120 µg) in the three-component blend attracted significantly large numbers of male moths (*F*_(9,30)_ = 19.31, *p*<0.001). Attraction of male *D. tabulaeformis* to the optimal three-component blend was unaffected by the addition of *Z*5-12:OAc and/or *Z*5-12:OH to that blend (Table 3).

**Table 3:** Catches of male *Dendrolimus tabulaeformis* in delta-shaped traps baited with various blends of potential pheromone components between 1 and 14 August 2011 in the experimental forest of Ping'quan city, He'bei province, China.

Composition of baits (µg)					Mean catch ± SE/trap^a^ *N* = 4
*Z*5,*E*7-12:OAc	*Z*5,*E*7-12:OH	*Z*5,*E*7-12:OPr	*Z*5-12:OAc	*Z*5-12:OH	
1600	—b	—	—	—	0.25±0.25b
—	1600	—	—	—	0
1600	1600	—	—	—	0.25±0.25b
1600	1600	0.7	—	—	0.25±0.25b
1600	1600	7	—	—	1.25±0.25b
1600	1600	70	—	—	31.75±16.07a
1600	1600	70	200	—	26.50±5.12a
1600	1600	70	—	400	18.25±6.25a
1600	1600	70	200	400	34.75±12.27a
1600	1600	120	—	—	20.50±8.05a
Control					0.50±0.50b

a Means followed by the same letter in column are not significantly different at the 5% confidence level by Tukey's tests (*F*_(9,30)_ = 19.31, *p*<0.001).

b “—” indicate the components were not included.

**Figure 2:**
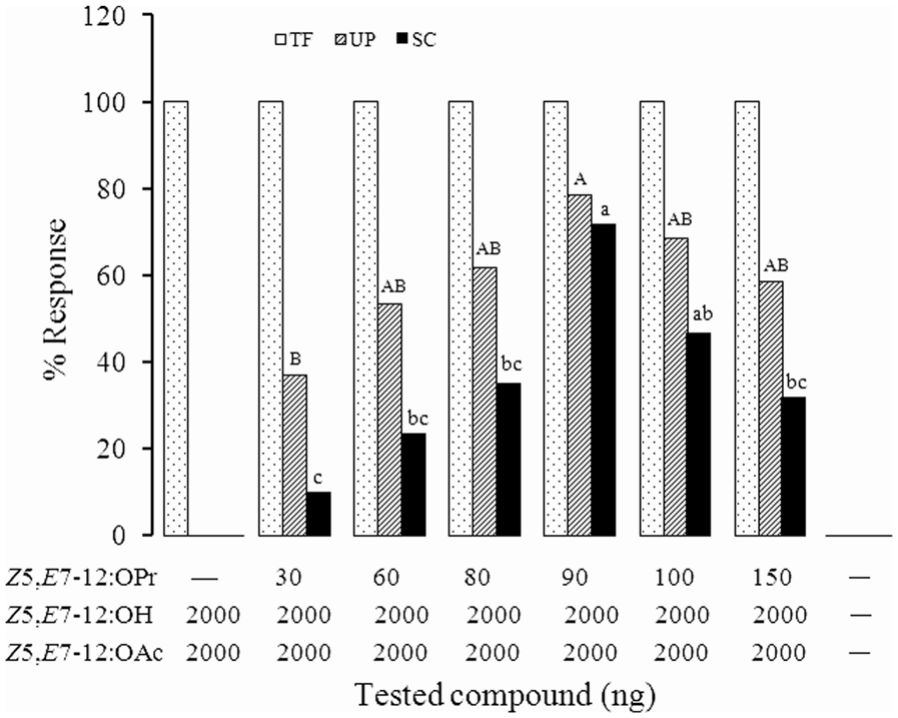
Percentages of males exhibiting three key behaviors in the flight tunnel in response to various doses of *Z*5,*E*7-12:OPr blended with fixed amounts of *Z*5,*E*7-12:OAc and *Z*5,*E*7-12:OH (doses in ng). Taking flight = TF, (light bars); upwind flight over a 1.2-m distance = UP, (hatched bars with different upper-case letters were significantly different by Tukey's test at *p* = 0.05 following ANOVA, *F*_5,12_ = 4.30, *p* = 0.018); and source contact = SC, (dark bars with different lower-case letters were significantly different by Tukey's test at *p* = 0.05 following ANOVA, *F*_5,12_ = 13.47, *p*<0.001).

In wind tunnel bioassays, all males tested took flight in response to all synthetic blends tested except the solvent control, indicating that even the incomplete blend of *Z*5,*E*7-12:OAc and *Z*5,*E*7-12:OH in a 1∶1 ratio was sufficient to elicit flight initiation (Figure 2). When the minor component, *Z*5,*E*7-12:OPr, was added to the two-component blend, male moths approached the pheromone source, and the highest proportion of males contacting the source occurred with a 2000∶2000∶90 of *Z*5,*E*7-12:OAc, *Z*5,*E*7-12:OH, and *Z*5,*E*7-12:OPr (72% of test males approached the source). In contrast, the 2000∶2000∶150 ng ratio significantly decreased source contacts compared to the 2000∶2000∶90 ratio (*F*_(5,12)_ = 13.47, *p*<0.001).

## Discussion

The five components from the pheromone glands that elicited EAD responses were unambiguously identified as *Z*5-12:OAc, *Z*5-12:OH, *Z*5,*E*7-12:OAc, *Z*5,*E*7-12:OPr, and *Z*5,*E*7-12:OH by GC, GC-MS, GC-EAD analyses, and microscale derivatizations. Moreover, antennae of males responded to the *Z*5,*E*7-isomer of the conjugated dienes and the *Z*5-isomer of the monoenes acetate, alcohol, and/or propionate in EAD analyses, and the other isomers were not detected in the gland extracts. In wind tunnel bioassays, a mixture of the two major components *Z*5,*E*7-12:OAc and *Z*5,*E*7-12:OH in a 1∶1 ratio was sufficient to induce flight by males, but the minor component, *Z*5,*E*7-12:OPr was essential for optimal upwind flight and source contact. In total, our data suggest that the female-produced sex pheromone of *D. tabulaeformis* is a three-component blend of *Z*5,*E*7-12:OAc, *Z*5,*E*7-12:OH, and *Z*5,*E*7-12:OPr, and the optimum ratio has been determined to be ca. 100∶100∶4.5, which is close to the ratio found in analyses of the volatiles emitted by calling females (100∶100∶8). Furthermore, our data showed that male *D. tabulaeformis* strongly discriminate against off-ratio pheromone blends, which would impose strong stabilizing selection on the pheromone signal.

Species-specific pheromone blends can be generated from different sets of constituents, or by mixing the same set of constituents in different ratios [12]. The identified sex pheromones of *Dendrolimus* species share a common chemical theme of being C_12_ 5,7-dienes with alcohol, acetate, propionate, or aldehyde functional groups, with species-specific blends being generated by using different subsets of components and/or ratios of components. For example, in the species studied here, a specific ratio of sex pheromone components is required in order to achieve optimal male flights. However, the best attractant for male *D. spectabilis*, which is sympatric with *D. tabulaeformis* in Shan'dong, He'bei, Liao'ning, and Inner Mongolia, consists of a 100∶3∶25 blend of *Z*5,*E*7-12:OH/*Z*5,*E*7-12:OAc/*Z*5,*E*7-12:OPr [6], with *Z*5,*E*7-12:OH as the principal sex pheromone component [2]. In contrast, comparative studies of the sex pheromones of the most closely related species, *D. punctatus*, which is sympatric with *D. tabulaeformis* in Shăn'xi, He'nan, and Chong'qing, suggest that male moths of this species respond to a broad range of ratios of *Z*5,*E*7-12:OH/*Z*5,*E*7-12:OAc/*Z*5,*E*7-12:OPr blend (median blend 100∶82∶30) [13]. *Dendrolimus punctatus*, *D. spectabilis*, and *D. tabulaeformis* all share the same three pheromone components, *Z*5,*E*7-12:OH, *Z*5,*E*7-12:OAc, and *Z*5,*E*7-12:OPr, but the differences in the ratios of the three-component blends are essential for maintaining separate communication channels.

Furthermore, the bioassay data suggests that *Z*5-12:OAc and *Z*5-12:OH are not part of the attractive pheromone blend of *D. tabulaeformis*, although they elicit relatively strong EAD responses from male antennae, because neither of these compounds enhanced the attractiveness of three-component blend in field test. The same phenomenon was also observed in *D. punctatus* [13]. However, *D. tabulaeformis* is partially sympatric with *D. kikuchii* in He'nan province, and a major difference between the two pheromone systems is that the pheromone blend of *D. kikuchii* substitutes *Z*5-12:OAc for *Z*5,*E*7-12:OPr in an optimal ratio of 100∶20∶25 *Z*5,*E*7-12:OAc/*Z*5,*E*7-12:OH/*Z*5-12:OAc [8]. Thus, the minor components Z5-12:OAc and *Z*5,*E*7-12:OPr may serve to enhance the specificity of the signals in *D. tabulaeformis* and *D. kikuchii*. In addition, the antennae of male *D. tabulaeformis* responded to synthetic *Z*5,*E*7-12:Ald and *E*5,*Z*7-12:Ald in our GC-EAD analyses, but the two compounds were not detected in pheromone gland extracts. It has been reported that the sex pheromones of *D. superans* and *D. houi* consist of *Z*5,*E*7-12:Ald [7] and *E*5,*Z*7-12:Ald [9], respectively. *Dendrolimus tabulaeformis* is broadly sympatric with *D. superans* in Inner Mongolia, He'bei, and Liao'ning, and sympatric with *D. houi* in Shăn'xi and Chongqing. Addition of *Z*5,*E*7-12:Ald (ca. 5% of *Z*5,*E*7-12:OAc) to the three-component blend of *D. tabulaeformis* resulted in decreased captures of males. Thus, these two aldehydes might play an important role in chemical communication of sympatric, closely related species, either as essential minor components of pheromone blends, or conversely as interspecific inhibitors.

The optimal blend of the sex pheromone components of *D. tabulaeformis* worked out in this study should find immediate use in monitoring this pest in Chinese forests.

## Materials and Methods

### Ethics statement

All necessary permits were obtained for the described field studies. The forestry bureau of Ping'quan city, responsible for Da'shi lake forest center, issued the permission for our field studies in the Chinese pine forest at this site. These field studies did not involve endangered or protected species.

### Insects

Cocoons of *D. tabulaeformis* were collected from the host tree, *Pinus tabulaeformis* at Da'shi lake forestry center near Ping'quan city, He'bei province on 30 July 2011 and maintained in a rearing room at 25±1°C, 60–80% relative humidity (RH) with a photoperiod of 16∶8 h light∶dark until adult emergence. Newly emerged males and females were housed separately under the same conditions. Virgin females were used for preparation of pheromone extracts, whereas males were used for EAG studies.

### Sample collection

Sex pheromone glands were excised from females during the peak period of calling (0–2 d old, 4–7 h into the scotophase) and extracted for 30 min with hexane (10 µl per gland). The glands were removed before sealing the extracts in glass tubes, which were stored at −20°C until bioassay and chemical analyses.

Pheromones emitted by 1–2 d old calling virgin females held in 0.02 m^3^ metal screen cages were collected by solid phase microextraction (SPME, 70 µm CarboWax divinylbenzene (CW/DVB) coated fiber, Supelco Inc., Bellefonte, PA, USA) under ambient laboratory conditions. The SPME fiber was placed 1–2 mm from the extruded gland, sampling from the onset of calling (about 3 h into the scotophase) to the end of scotophase.

### Chemicals and derivatives

All geometrical isomers of 5,7-dodecadien-1-yl acetate (*Z*5,*Z*7-, *E*5,*E*7-, *E*5,*Z*7-, and *Z*5,*E*7-12:OAc) and its corresponding alcohols and propionates, together with *Z*5-12:OAc and *Z*5-12:OH were purchased from Chemtech B. V. (Amsterdam, The Netherlands). Chemicals used as stimuli for electrophysiological and field trials were >98% chemically pure and >95% isomerically pure. C_14_-C_24_ straight-chain hydrocarbons for assigning Kováts retention indices (KIs) were purchased from TCI Co. (Tokyo, Japan). 4-Methyl-1,2,4-triazoline-3,5-dione (MTAD, 95%) was purchased from Aldrich Chemical (Milwaukee, WI, USA), and dimethyl-disulfide (DMDS, 99%) was purchased from Acros Organics (Geel, Belgium).

The positions of double bonds in conjugated dienes were determined by means of MTAD derivatization [14], [15], and in monounsaturated alkenes by the iodine-catalysed addition of dimethyl-disulfide across the double bond [16]. In this study, 30 female equivalents (FE) of extract were derivatized by MTAD and DMDS reagents respectively, as previously described [8], [16]. The derivatives were analyzed by GC-MS.

### Gas chromatography-electroantennographic detection (GC-EAD) analyses

The coupled GC-EAD system used was based on that described by Kong et al. (2011). The HP6890 GC (Agilent, Palo Alto, CA, USA) was equipped with a split/splitless injector, a DB-WAX column (J&W Scientific, Folsom, CA, USA, 30 m×0.25 mm i.d. ×0.25 µm) with the column effluent split between the FID and EAD detectors (FID/EAD, 1∶2). The effluent exited into a purified and humidified airstream (450 ml/min) directed over the antennal preparation. A detached antenna from a 1-to 3-d-old male with the terminal segment removed, was mounted between two EAG probes (PRG-2, Syntech, Kirchzarten, Germany) with electrically conductive gel (Spectra 360 electrode gel, Parker Laboratories Inc. Orange, NJ, USA). Synthetic compounds and SPME fibers with loaded samples were injected splitless using a temperature program starting at 60°C for 1 min, then programmed at 6°C/min to 230°C (15 min hold), using nitrogen carrier gas (33 cm/sec). After adsorption, the CW/DVB fiber was treated with a 1 µl aliquot of hexadecane, eicosane, and tetracosane (5 ng/µl of each in dichloromethane) with a syringe, and then immediately introduced into the HP 6890 GC injector where it was desorbed for 2 min (injector temperature 220°C). The purpose of these standards was to assign retention indices to the components of the crude extract [17]. Acquisition and analysis of the EAD signals were performed by GC-EAD software (16-bit version, Syntech, Kirchzarten, Germany).

### GC and GC-MS analyses

Quantitative analyses of 21 glands extracted individually were conducted on an HP7890 GC with an FID detector using a DB-WAX column (J&W Scientific, Folsom, CA, USA, 30 m×0.25 mm i.d. ×0.25 µm film) in splitless mode, temperature program, 60°C for 1 min, then 6°C/min to 240°C (hold 10 min). Injector and detector temperatures were set at 230°C and 250°C, respectively. A 1 µl aliquot of *E*5,*Z*7-12:OAc (5 ng/µl) was used as an external standard to quantify the pheromone components. Absolute amounts were calculated from the integrated peak area of each component relative to that of the external standard.

Pheromone gland solvent extracts, SPME collections (desorbed for 2 min in the injector), and references also were analyzed with a Finnigan Trace DSQ GC-MS (EI mode, 70 eV, mass range 41–560 amu) on DB-WAX and DB-5MS capillary columns (splitless mode, 30 m×0.25 mm i.d. ×0.25 µm film) under the following conditions: injector, ion source, and transfer line temperatures were held at 220°C, 250°C, and 240°C, respectively; helium as carrier gas at 1.0 ml/min; scan time 0.2 sec; oven temperature program: 60°C for 1 min, then 6°C/min to 260°C, hold for 20 min. When analyzed on the DB-5MS column, a 1 µl aliquot of a series of straight-chain aliphatic hydrocarbons (from C_14_-C_18_, 5 ng/µl of each in dichloromethane) was added directly to the sample-loaded fiber or solvent extracts with a syringe in order to assign retention indices to the components of crude extracts. DMDS and MTAD derivatives of pheromone gland extracts were analyzed with the same GC-MS (EI mode, 70 eV, mass range 41–560 amu) on a DB-5MS capillary column (30 m×0.25 mm i.d. ×0.5 µm film) in splitless mode. The column temperature was programmed from 80°C for 1 min, then 8°C/min to 280°C (10 min hold). Injector, ion source, and transfer line temperatures were set at 280°C, 250°C, and 250°C, respectively. In GC-MS analysis, compounds were identified by comparison of retention indices and mass spectra with those of synthetic standards or their derivatives.

### Behavioral assays

Field bioassays were conducted in the Da'shi lake forestry center near Ping'quan city, He'bei province. At 20–30 m intervals, delta-shaped traps made from adhesive cardboard were suspended from trees 1.5–1.7 m above ground in randomized complete blocks. In each of four blocks (replicates), traps were baited with a gray sleeve septum (The West Company, Phoenixville, PA, USA) impregnated with test chemicals in hexane, and 200 µg of BHT in 100 µl CH_2_Cl_2_ were added subsequently as an antioxidant. The experiment started on 1 August 2011 and was terminated two weeks later.

Synthetic compounds were selected for more detailed examination in a wind tunnel (flight section 220×100×120 cm) based on data from the field tests. Air purified by filtration through activated charcoal was blown by a centrifugal fan at 40 cm/s into the tunnel and the outgoing air was exhausted with a second fan. The flight section was lit diffusely from the top at 2–3 lx with a red light, and the room was kept at 25±2°C and 50–65% RH. Synthetic compounds in different ratios were released from the centre of the upwind end of the tunnel by means of gray rubber septa. Males 1–3 days of age were placed in the tunnel room at the initiation of scotophase, 3 h prior to testing, to acclimate to the conditions of the assay room. Males were scored for three key behaviors in the flight sequence: taking flight (TF), initiation of upwind flight (UP), and source contact (SC) [18]. During each 4 h test period, two treatments were tested, with twenty males tested individually per treatment. The two- and three-component blends were prepared in hexane and applied in 50 µl aliquots to gray rubber septa to achieve increasing dosages of 0, 30, 60, 80, 90, 100, and 150 ng/septum for *Z*5,*E*7-12:OPr while keeping the major components, *Z*5,*E*7-12:OH and *Z*5,*E*7-12:OAc in a 1∶1 fixed ratio (2000 ng/septum for each) in order to determine the behavioral effect of variable amounts of the minor component *Z*5,*E*7-12:OPr.

### Statistical analysis

Trap-catch data from field bioassays were subjected to transformation (log(x+1)) to ensure normal distribution and homogeneity of variance and then analyzed by one-way ANOVA followed by Tukey's test with a significance level of 0.05, for comparisons of means (SPSS13.0). The zero trap-catch data were not included in the analyses because of their lack of variance. For wind tunnel bioassays, the percentage of males responding in the flight tunnel was arcsin(x) transformed and then submitted to a one-way ANOVA, followed by Tukey's test with a significance level of 0.05, for comparisons of means.
